# Balancing Organic and Inorganic Carbon Dynamics in Enhanced Rock Weathering: Implications for Carbon Sequestration

**DOI:** 10.1111/gcb.70186

**Published:** 2025-04-16

**Authors:** Kaiyu Lei, Franziska B. Bucka, Pedro P. C. Teixeira, Franz Buegger, Christopher Just, Ingrid Kögel‐Knabner

**Affiliations:** ^1^ Chair of Soil Science, School of Life Science Technical University of Munich Freising Germany; ^2^ Soil Geography and Ecosystem Research, Institute of Physical Geography Goethe University Frankfurt Frankfurt am Main Germany; ^3^ Research Unit Environmental Simulation Helmholtz Zentrum München, German Research Center for Environmental Health Neuherberg Germany; ^4^ Institute for Advanced Study Technical University of Munich Garching Germany

**Keywords:** ^13^C isotopic tracing, basalt weathering, carbon sequestration, inorganic carbon formation, organic and inorganic carbon pools, organic carbon turnover, rising pH, soil carbon fluxes

## Abstract

Enhanced rock weathering (ERW) is a promising strategy for CO_2_ removal via promoting inorganic carbon (IC) sequestration. However, knowledge gaps persist regarding its influence on the largest terrestrial carbon pool, soil organic carbon (SOC) and how these effects evolve as weathering progresses. This study investigated how basalt weathering influences soil carbon fluxes and organic matter (OM) turnover. Over a 6th‐month incubation, we applied fresh basalt (fine‐sized, olivine‐rich) and weathered basalt (coarse‐ and fine‐sized, olivine‐depleted) to temperate cropland topsoil, incorporating with ^13^C‐labelled straw. Fresh basalt increases soil pH via rapid H^+^ neutralization during olivine dissolution, releasing soluble Mg^2+^ and increasing bicarbonate alkalinity. Combined with continuous carbonic acid dissociation for olivine dissolution, they synergistically enhance dissolved inorganic carbon (DIC) accumulation in soil solution and effluent (~0.4%), promoting soil inorganic carbon (SIC) accrual via carbonate precipitation (~4%). However, rising pH concurrently induces significant SOC losses (~17%), resulting in net C losses of ~13%. As basalt weathering progresses (olivine‐depleted), slower H^+^ neutralization and carbonic acid dissociation during less‐reactive Ca‐bearing mineral dissolution stabilize soil pH, limiting DIC formation. The released Ca^2+^ prioritizes SIC accrual via Ca‐carbonate precipitation (~4%). Meanwhile, higher specific surface area (SSA) and exchangeable Ca^2+^ enhance retention and stabilization of both native and straw‐derived OC, reducing net C losses (~6%). At both weathering stages, over 95% of total C remaining in soils and effluent exists in organic form. Straw inputs acidify soils by releasing additional free H^+^ during decomposition, competing with carbonic acid for olivine dissolution and reducing bicarbonate alkalinity, which limits the DIC and SIC accrual at both weathering stages. Since soils continuously receive OM input, understanding the balance between these interactive processes is crucial for optimizing long‐term carbon sequestration strategies. Therefore, sustaining SOC by minimizing SOC losses should be prioritized for long‐term carbon sequestration, besides IC accrual for ERW, particularly as weathering progresses.

## Graphical abstract

Enhanced rock weathering involves adding crushed silicate rock to soils, capturing atmospheric carbon dioxide by forming inorganic carbon. Despite significant inorganic carbon accumulation at the initial stage, this method can also trigger significant losses of soil organic carbon due to increased soil pH. However, with minerals weathering, they become more effective at retaining organic carbon besides inorganic carbon formation. Importantly, plant residue inputs can limit inorganic carbon formation by acidifying the soil. Our findings emphasize that minimizing soil organic carbon losses is crucial for optimizing the long‐term climate benefits of enhanced rock weathering.

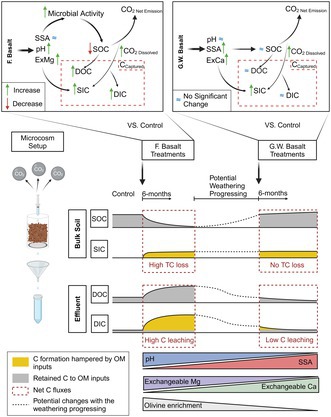

## 1 Introduction

To mitigate the escalating climate crisis, enhanced rock weathering (ERW) has emerged as a promising strategy for atmospheric carbon dioxide (CO_2_) removal. Initially proposed in 1990 (Seifritz 1990), ERW has gained substantial attention over recent decades as an effective strategy for CO_2_ sequestration (Beerling et al. 2020; Buss, Hasemer, Ferguson et al. 2024; Buss, Hasemer, Sokol et al. 2024; Hartmann et al. 2013; Kantola et al. 2017; Kelland et al. 2020; Köhler et al. 2010; Schuiling and Krijgsman 2006; Sokol et al. 2024; Wang and Kuzyakov 2024; Xu et al. 2024). This method involves applying finely ground calcium (Ca) and magnesium (Mg)‐rich silicate mineral powders to soils, which accelerates the neutralization of H^+^ derived from carbonic acid dissociation during silicate mineral dissolution (Oelkers et al. 2018). This results in an increased dissolved inorganic carbon (DIC) concentration in soil solution as bicarbonate, and releases soluble ions (e.g., Ca^2+^ and Mg^2+^), which can be transported to aquatic systems for long‐term sequestration (Oelkers et al. 2018; Clarkson et al. 2024; Hasemer et al. 2024). Under alkaline conditions, these ions can precipitate with DIC as stable inorganic carbon (IC) in the form of Ca/Mg carbonates in the soil (SIC) or aquatic systems (Clarkson et al. 2024; Hasemer et al. 2024). Among various silicate minerals, basalt, as an abundant, cost‐effective, and fast‐weathering rock enriched with Mg and Ca, is regarded as particularly suitable for large‐scale ERW applications (Buss, Hasemer, Sokol et al. 2024).

Free H^+^ neutralization in soil solution during olivine dissolution results in an increased soil pH. The pH elevation associated with ERW could affect soil organic carbon (SOC) content, as evidenced by SOC losses observed in soils treated with lime (Paradelo et al. 2015). Rising pH can stimulate microbial and enzymatic activities (Wang and Kuzyakov 2024; Zhang et al. 2020) and increase nutrient availability (Kantola et al. 2017), potentially accelerating soil organic matter (SOM) decomposition and leading to SOC losses. Extrapolated from this, the increased pH caused by ERW materials may also affect SOC in the long term. Yet, the magnitude and long‐term persistence of these effects remain uncertain, especially given the variability in the weathering stages of ERW materials (Buss, Hasemer, Ferguson et al. 2024; Vicca et al. 2022).

In addition to alkalinity effects, recent studies have reported positive (Bi et al. 2024; Buss, Hasemer, Ferguson et al. 2024; Xu et al. 2024), neutral (Bucka et al. 2024; Sokol et al. 2024) and negative effects (Klemme et al. 2022) of ERW materials on SOC accrual. Theoretically, ERW can enhance carbon sequestration by promoting the formation of mineral‐associated organic matter (MAOM), driven by the release of reactive secondary minerals and facilitating cation bridging during silicate mineral weathering, which has been observed in recent studies using fresh wollastonite (Xu et al. 2024). In contrast, a field trial applying meta‐basalt to calcareous soils reported reduced MAOM‐C, attributed to decreased active microbial biomass associated with ERW (Sokol et al. 2024). However, previous studies used only fresh ERW materials, which typically have a lower specific surface area (SSA) and fewer reactive sites. Thus, the net carbon sequestration potential of ERW materials at more advanced weathering stages remains uncertain. In addition, organic matter (OM) coating/aggregation on mineral surfaces and organic acid released during OM decomposition can significantly influence primary/secondary mineral weathering (Drever and Stillings 1997; Ritschel et al. 2023). Current ERW models, which primarily rely on theoretical oxide dissolution kinetics (Renforth 2019), may not adequately capture these complex interactions between OM dynamics and mineral dissolution processes. Thus, addressing these uncertainties is essential to optimize the implementation of ERW and accurately estimate its long‐term carbon sequestration potential.

Soils continuously receive OM inputs from plant residues, animals, and anthropogenic organic fertilizers. These inputs can significantly influence soil carbon dynamics, making it critical to account for the cycling and turnover of these new OM inputs when evaluating the effectiveness of ERW strategies. Although a few recent studies (Kelland et al. 2020; Xu et al. 2024) have offered some preliminary insights into how plant biomass inputs influence ERW‐driven changes in SOC content, they do not clarify the effects of ERW on both native and new OC as weathering progresses. Consequently, the mechanistic understanding of how these new and native OC pools interact with ERW‐induced IC pools remains incomplete, which makes the comprehensive evaluation of long‐term C sequestration by ERW impossible.

To address these knowledge gaps, we conducted a microcosm experiment applying three basalt materials at two different weathering stages and particle sizes from the same quarry to a temperate Cambisol topsoil sampled from the same region in Germany. These basalts simulated the progression of basalt weathering. To simulate natural OM inputs, we also applied ^13^C‐labeled straw, enabling us to quantify the distinct fate and stabilization mechanisms of new OC and native OC. We aim to answer the following key questions: (a) Which mechanisms drive C stabilization and losses in soils applied with basalt materials at different weathering stages? (b) What are the long‐term implications of basalt application for soil carbon sequestration? (c) How do interactions between SOC and IC pools influence the overall effectiveness of ERW as a climate mitigation strategy? By addressing these questions, this study aims to clarify the net impacts of ERW on the cycling and interactions of OC and IC at different weathering stages by providing a complete carbon flux assessment.

## 2 Materials and Methods

### 2.1 Materials and Incubation Setup

Approximately 250 g of dried agricultural soil (< 2 mm) was incubated in microcosms (5 cm in height, 8 cm internal diameter) for 6 months in a dark, open system at a constant room temperature of 25°C. It was incubated in darkness to minimize the photodegradation of OM and simulate subsoil conditions. The soil was sourced from the Ap horizon of the cropland on the slope of Süssenbach (49°06′12″ N, 12°21′37″ E) in the Falkensteiner Vorwald region, part of the Bavarian Forest, Southeast Germany. The region experienced an average annual temperature of 8.9°C and annual precipitation of 875 mm. The soil parent material is Regensburg crystal granite, and it is classified as Cambisol according to IUSS Working Group WRB (2022). A detailed description of the soil can be found in Lei et al. (2025).

Basalt and weathered basalt materials were sourced from the Zeilberg quarry (50°11′46″ N, 10°40′27″ E) managed by Basalt‐Actien‐Gesellschaft, Germany. Fresh basalt (enriched in olivine) was obtained from ~180 m depth, while weathered basalt (depleted in olivine) came from a post‐mined area exposed to the natural environment for over 8 years. Weathered basalt was crushed to < 2 mm. A subsample was ground (20 Hz, 5 min, Vibrating Mill MM 400, Retsch, Germany) to silt‐size particles with a comparable particle size to fresh basalt. The ball milling tank was cooled with liquid nitrogen. The grinding procedure was carried out at intervals to prevent overheating. Materials were mixed with soils at a 4% weight ratio.

Qualitative X‐ray diffraction (XRD) analysis (Figure S1) and total element analysis using inductively coupled plasma optical emission spectrometry (ICP‐OES) (Table S1) were carried out to estimate the olivine mineral enrichment at two weathering stages. As the olivine is the mineral enriched with Mg and Fe, the qualitative XRD with distinct olivine peaks and different total Mg and Fe from total element analysis can demonstrate the degree of olivine enrichment in Fresh basalt and Weathered basalt materials. Detailed properties of these materials and XRD results are shown in Table S1 and Figure S1.

After cooling with liquid nitrogen, the ^13^C‐labelled rice straw was homogenized by ball‐grinding for 1.5 min at 25 Hz. It was mixed at 0.8% by weight with soil and treatments. Straw had OC content at 400 mg/kg with a C/N ratio of 14 and a ^13^C enrichment of 238‰. Additional information on the straw is shown in Table S1.

To keep the dosage comparable to other field experiments (Kantola et al. 2017; Vienne et al. 2022), the dosage of treatments is 5 kg/m^2^ at 10 cm depth (equal to 4%). The microcosm experiment included one control and 5 treatments: +F. Basalt (96% soil, 4% fresh basalt), +W. Basalt (96% soil, 4% weathered basalt), +G.W. Basalt (96% soil, 4% ground‐weathered basalt), +Straw (99.2% soil, 0.8% rice straw), +F. Basalt & Straw (95.2% soil, 0.8% rice straw, 4% fresh basalt), +W. Basalt & Straw (95.2% soil, 0.8% rice straw, 4% weathered basalt) and +G.W. Basalt & Straw (95.2% soil, 0.8% rice straw, 4% ground‐weathered basalt). Each of them had five replicates in a randomized block design. Corrections were made for the dilution effect caused by the additional treatments (4%). The physicochemical properties of these initial mixtures are shown in Table S2.

In each microcosm, soils were first saturated to determine water‐holding capacity and then pre‐incubated for 3 days. To simulate an annual precipitation of approximately 875 mm, a weekly irrigation of 84 mL of distilled water was applied, generating effluent for analysis. Between the weekly irrigations, additional distilled water was carefully dispensed to maintain soil moisture above 50% of its water‐holding capacity, without producing effluent. All water was added using an 80 mL syringe to minimize surface sealing, and the microcosms were weighed before each irrigation to track soil moisture content.

### 2.2 Soil Sampling and Physicochemical Analysis

After 6 months of incubation, the microcosms were carefully sectioned vertically into halves. Half of each section was further divided into top (0–0.5 cm), middle (0.5–4.5 cm) and bottom (4.5–5 cm) layers. Only the middle section was used for further analysis, as the top layer was potentially affected by watering turbulence, and the bottom layer might have additional sedimentation materials.

The middle section of the soil was oven‐dried at 40°C, with macroaggregates gently broken down and sieved to < 2 mm for the analysis of Brunauer–Emmett–Teller (BET) surface areas (referred to as SSA), particle size distribution, pH, effective cation exchange capacity (eCEC) and exchangeable cations. Subsamples were ball‐milled for 1 min at 25 Hz to homogenize them for TC, SOC, SIC, total elements and δ^13^C analysis.

The SSA was measured using nitrogen (N_2_) adsorption at 77 K following the BET method (Brunauer et al. 1938) by the gas adsorption analyzer (AUTOSORB I, Quantachrome, USA). Approximately 2 g of dry soil was prepared for the measurement. To ensure accuracy and remove moisture, the soil samples were outgassed overnight under vacuum conditions with helium at 40°C. The detailed data is shown in Table S3.

For particle size distribution, ~10 g of soil was moistened and dispersed using 0.05 M L^−1^ sodium pyrophosphate with an energy input of 450 J L^−1^. Sand‐size fractions (> 63 μm) were separated by wet sieving, while the < 63 μm fraction was further dispersed overnight and analyzed using X‐ray granulometry (SediGraph III Plus, Micromeritics, USA). The detailed data is shown in Figure S2 and Table S3.

For pH analysis, ~5 g of soil was mixed at a 1:2.5 (w/v) ratio with 0.01 M L^−1^ calcium chloride solution and measured with the pH meter (Seven Easy pH, Mettler‐Toledo, Switzerland).

For exchangeable cations and eCEC, the cobalt hexamine method was employed for its repeatability in slightly acidic soils (Nel et al. 2023). In brief, ~ 5 g of soil was mixed with a 0.0166 M L^−1^ cobalt hexamine solution and left to react overnight. The mixture was centrifuged at 1610 g force for 10 min, with the supernatant filtered through Mushell filter paper (Ahlstrom Munksjo) and analyzed using ICP‐OES.

Total elements of soils and basalt materials were measured via ICP‐OES after digestion with the mixture of HClO_4_‐HNO_3_‐HF‐HCl (Page 1982).

### 2.3 Soil Respiration Measurements

The CO_2_ respiration was measured in a closed chamber system (Pumpanen et al. 2004) using the titration method (Eco Titrator, Metrohm AG, Switzerland). The microcosms were incubated overnight at 25°C in a closed chamber with a beaker containing 15 mL of 0.1 M NaOH, which absorbed the emitted CO_2_. To stabilize the adsorbed CO_2_, 0.5 M BaCl_2_ was added. A control microcosm consisting of ~250 g of pure quartz was run in parallel to determine baseline OH^−^ consumption. By subtracting the unused OH^−^ in the controls from that in soil‐containing microcosms, the CO_2_ emission from each sample was obtained. Measurements were conducted twice weekly (starting from Day 3) for the first month, then reduced to weekly, biweekly and monthly intervals starting from the third month. For each interval (e.g., Day 3–5, Day 5–7), CO_2_ flux rates were converted to cumulative emissions via the trapezoidal rule, and these intervals were summed to estimate the total CO_2_ released. The pH threshold for carbonate analysis was set at 8.3. Prior to titration, pH was adjusted using standard buffer solutions.

### 2.4 Effluent Measurements

Effluent was collected weekly and immediately sealed with caps and Parafilm. Samples were stored at 4°C before analysis. DIC concentration was measured within 48 h via titration with 0.1 M H_2_SO_4_ to pH 4.5 using the titration method (Eco Titrator, Metrohm AG, Switzerland). Prior to titration, pH was adjusted using standard buffer solutions.

Effluent was filtered through 0.45 μm PES filters for dissolved organic C (DOC), metal and heavy metal analysis. DOC was quantified with a TOC analyzer (Shimadzu TOC‐5050A). The Ca, Mg, Cd, Ni and Cu concentrations were measured by ICP‐OES. Detailed data is shown in Table S2.

### 2.5 TC, SOC, SIC, δ^13^C Analysis and C Balance Calculation

TC, SOC, SIC, and δ^13^C (‰ V‐PDB) contents of the soil were measured via an isotopic ratio mass spectrometer (IRMSdelta V Advantage, Thermo Fisher, Dreieich, Germany) coupled with an Elemental Analyzer (Euro EA, Eurovector, Milan, Italy). Carbonates were removed from soils using 2 N HCl for SOC content and δ^13^C measurements (Midwood and Boutton 1998), while non‐acid‐treated soils were analyzed for TC and δ^13^C. SIC is calculated by subtraction between TC and SOC.

The proportion of straw‐derived OC stabilized (*f*) in the soil was calculated using the equation:

$$f=\frac{\delta_{t}-\delta_{s}}{\delta_{r}-\delta_{s}}$$

where δ_*t*_ = δ^13^C of the soil with additional treatments after carbonate removal, δ_*s*_ = δ^13^C of their respective control soils after carbonate removal; δ_*r*_ = δ^13^C of the added treatments.

Over 6 months of incubation, CO_2_ emissions and DIC/DOC in the effluent measured at multiple time points were integrated using the trapezoidal rule to derive the cumulative release of CO_2_‐C, DIC and DOC. At the end of the incubation, SOC and SIC content were measured to estimate C retaining in soils. Summarizing the final soil C (SOC + SIC) with the cumulative CO_2_‐C, DIC and DOC provided the C balance relative to the initial soil TC. The IC pool in the system is defined as the summary of SIC and DIC. The C recovery was determined by comparing the sum of final C pools (SOC, SIC, CO_2_‐C, DIC, DOC) to the initial soil TC.

### 2.6 Statistical Analysis

The statistical analysis was conducted in R (version 4.3.1) (R Core Team, R 2013), using packages including ‘ggplot2’ (Bogovic et al. 2016), ‘paletteer’ (Hvitfeldt 2021), ‘patchwork’ (Pedersen 2024), ‘multcomp’ (Hothorn et al. 2016) ‘ggdist’ (Kay 2023), ‘dplyr’ (Wickham et al. 2020), ‘tidyr’ (Wickham and Henry 2020), ‘sp’ (Bivand et al. 2013), ‘randomForest’ (Breiman 2001), ‘caret’ (Kuhn et al. 2020).

To compare the significance of TC content, SOC content, SIC content, pH, eCEC, exchangeable Ca, exchangeable Mg, straw‐derived OC content, native SOC content, and C fluxes between treatments, a two‐way ANOVA was conducted to perform pairwise comparisons at a significance level of 0.05, followed by a Tukey HSD test for post hoc analyses. The Levene Test and Shapiro–Wilk Test were performed to validate dataset homogeneity and assess the normality of residuals in the model, respectively.

To better select relevant variables for SOC and SIC contents prediction via the Random Forest model by reducing redundancy and identifying potential multicollinearity, Principal Component Analysis (PCA) was performed to evaluate the relationships among explanatory factors. After PCA analysis, the Random Forest model was employed to evaluate the factors influencing SOC and SIC contents, as well as their explanatory power. To optimize model performance, K‐fold cross‐validation was used to tune parameters, including the number of variables considered at each split. Model performance was assessed using out‐of‐bag (OOB) error and root‐mean‐square error (RMSE). The variable importance derived from the Random Forest model was analyzed to identify and rank the most influential explanatory factors for SOC and SIC contents.

## 3 Results

### 3.1 Soil C Contents After 6 Months of Incubation

**FIGURE 1:**
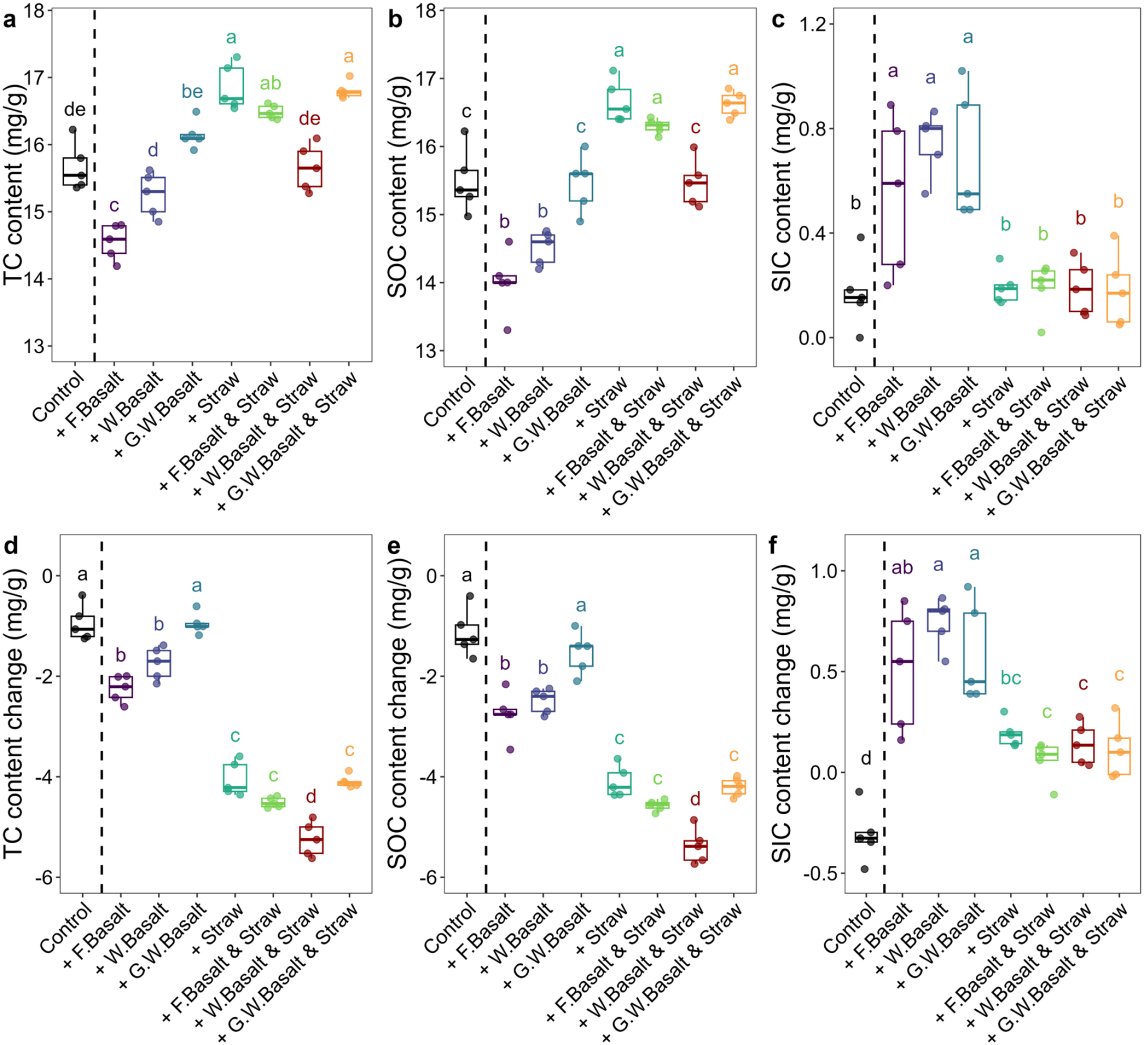
The carbon content and changes in soils after 6 months of incubation. (a) Total carbon (TC), (b) soil organic carbon (SOC) and (c) soil inorganic carbon (SIC) content in soils, and their respective changes (d), (e), (f) compared to the original mixture before their respective incubation period. Data was corrected for dilution effect, *n* = 5. The fresh basalt, weathered basalt and ground‐weathered basalt are referred to as ‘F. Basalt’, ‘W. Basalt’ and ‘G.W. Basalt’ in the figure, respectively.

After 6 months of incubation under lab‐controlled conditions, TC content in all treatments decreased significantly (Figure 1a). Fresh basalt treatments showed the greatest TC reduction (up to 14%; Figure 1d), while weathered and ground‐weathered basalt treatments remained comparable to controls (Figure 1a). With straw addition, the most significant TC reduction was observed in the weathered basalt treatments (~25%), which showed no difference compared to the control soils (Figure 1a). Other treatments with additional straw, including fresh basalt and ground‐weathered basalt treatments, exhibited TC contents similar to treatments with only straw. Overall, treatments with additional straw showed higher TC contents than the control soils (Figure 1a,d).

A similar pattern was observed for SOC content (Figure 1b). The fresh basalt and weathered basalt treatments both had significantly lower SOC content compared to the control soils, which were 10% and 8% lower by mean, respectively. The ground‐weathered basalt treatments showed SOC content comparable to the control soils (Figure 1b,e). Significant accumulations of SIC were observed in all treatments without straw addition, including fresh basalt, weathered basalt and ground‐weathered basalt treatments (Figure 1c). However, when straw was added, no significant difference in SIC content was observed between the control soils and any other treatments (Figure 1c,f).

In soils receiving straw, both TC and SOC declined significantly after 6 months of incubation (Figure 1d,e) without any SIC accumulation.

### 3.2 Soil Physicochemical Properties After 6 Months of Incubation

**FIGURE 2:**
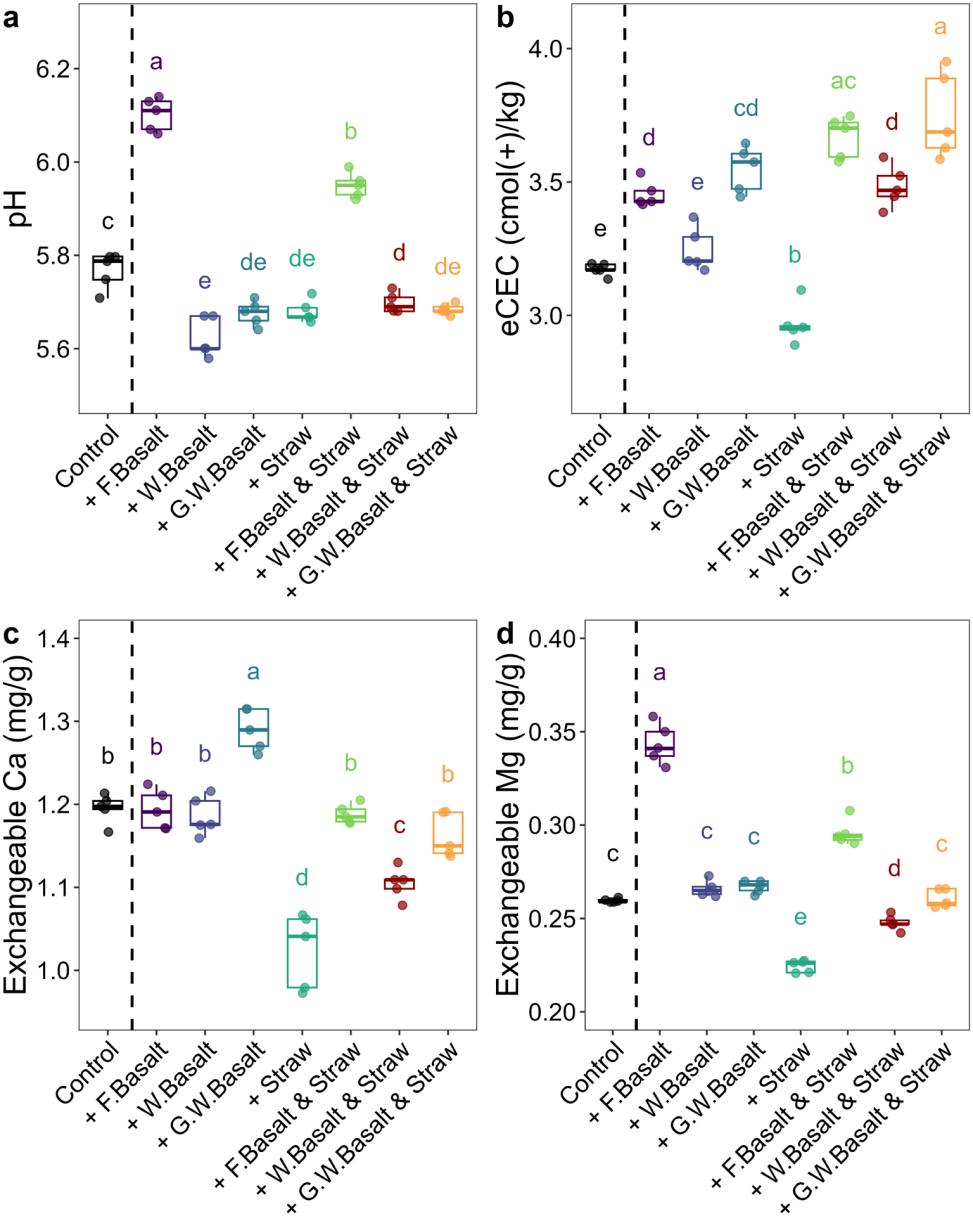
The chemical properties of the soil. (a) pH (CaCl_2_), (b) eCEC, (c) exchangeable Ca^2+^ and (d) exchangeable Mg^2+^ in soils after 6 months of incubation. Data was corrected for dilution effect, *n* = 5. The fresh basalt, weathered basalt and ground‐weathered basalt are referred to as ‘F. Basalt’, ‘W. Basalt’ and ‘G.W. Basalt’ in the figure, respectively.

Both the fresh basalt treatments and their mixture with straw significantly increased the pH, eCEC and exchangeable Mg^2+^ compared to the control soils (Figure 2a,b,d). No significant differences in exchangeable Ca^2+^ were observed between these treatments (Figure 2c). In contrast, the weathered and ground‐weathered basalt treatments, as well as their mixture with straw, showed a marginal change in pH (less than 0.1). Higher exchangeable Ca^2+^ in the ground‐weathered basalt treatments and higher exchangeable Mg^2+^ in the fresh basalt treatments contributed to a significant eCEC increase and elevated soluble Ca^2+^ and Mg^2+^ (Figure S5). However, adding straw alone reduced eCEC due to significant decreases in both exchangeable Ca^2+^ and Mg^2+^ (Figure 2b–d).

There were no significant differences regarding the particle size distribution between the solo treatments and the control soils after 6 months of incubation (Figure S2). Likewise, no significant differences were found between initial mixtures and mixtures after 6 months of incubation. No significant differences were found in the particle size distribution between treatments (Figure S2, Table S3).

The SSA, measured as BET surfaces, was similar between the fresh basalt treatments and the control soils (Table S1). The addition of weathered basalt and ground‐weathered basalt significantly increased the SSA compared to the control soils (Table S3), averaging 10 and 9.5 m^2^/g, respectively. Although adding straw slightly lowered the SSA across all treatments, only the weathered basalt treatments with straw exhibited a statistically significant decrease compared with the weathered basalt treatments alone (Table S3).

### 3.3 Soil δ^13^C Analysis Results

**FIGURE 3:**
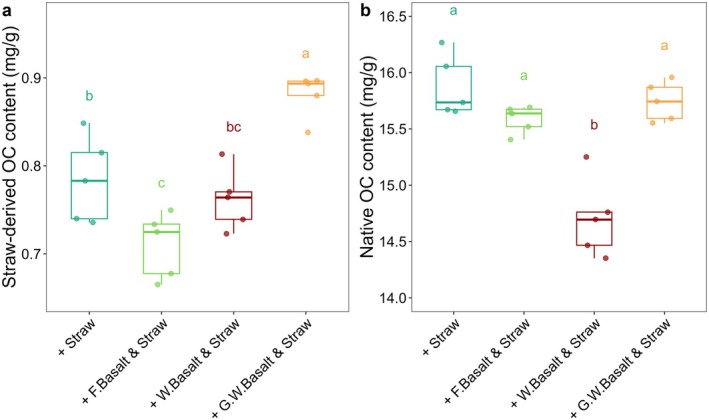
(a) Straw‐derived organic carbon (OC) content, and (b) native OC in soils based on δ^13^C‐OC in each treatment. The calculation equation was detailed in the Method section. Data was corrected for dilution effect, *n* = 5. The fresh basalt, weathered basalt and ground‐weathered basalt are referred to as ‘F. Basalt’, ‘W. Basalt’ and ‘G.W. Basalt’ in the figure, respectively.

The δ^13^C analysis revealed differences in C sources between treatments with and without straw addition (Figure 3). Over 75% of the straw‐derived OC in all treatments was lost from soils after 6 months of incubation. Soils treated with ground‐weathered basalt had a significantly higher straw‐derived OC content than the control soils and other treatments, whereas the weathered basalt treatments contained the lowest native SOC content. No significant differences were observed between the straw addition alone and its mixture with fresh basalt (Figure 3). Detailed soil δ^13^C data after 6 months of incubation are shown in Table S3.

### 3.4 Random Forest Model for Explaining SOC and SIC Content

**FIGURE 4:**
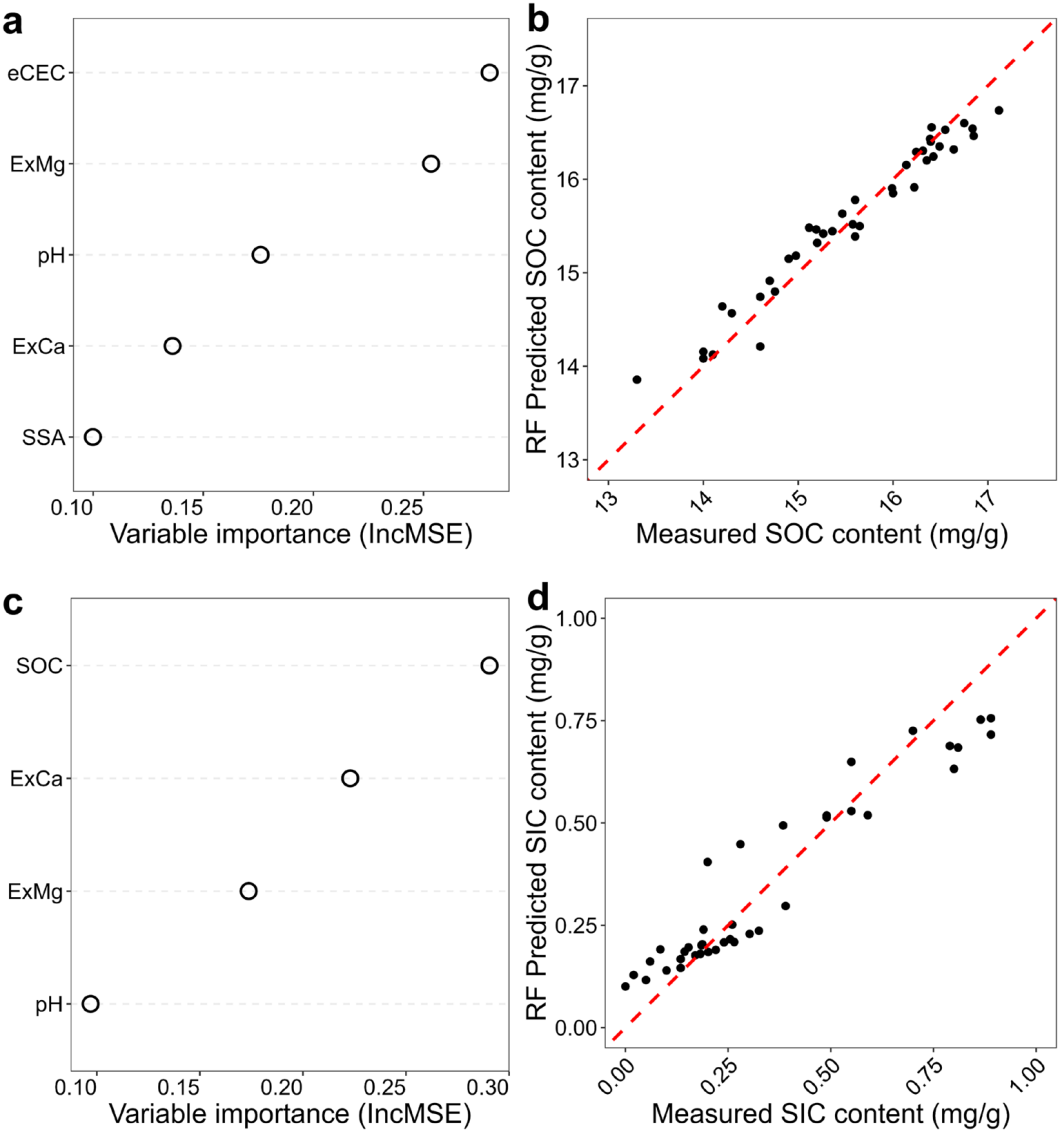
Random Forest model prediction of soil organic carbon (SOC) and soil inorganic carbon (SIC) content. The IncMSE was used to evaluate the variable importance for (a) SOC and (c) SIC content. The 1:1 line of predicted and measured content is marked as red dashed line in (b) and (d). The ‘ExCa’, ‘ExMg’ and ‘SSA’ are referred to the ‘exchangeable Ca^2+^’, ‘exchangeable Mg^2+^’ and ‘specific surface area’, respectively. Detailed statistical results are shown in Table S4.

The PCA analysis identified pH, eCEC, SSA, exchangeable Ca^2+^ and exchangeable Mg^2+^ as key variables influencing SOC and SIC contents (Figure S3). These variables were used in a Random Forest model, explaining over 75% of the variance in SOC content and over 55% in SIC content, with RMSE < 0.20 for both (Table S4). Among the variables, eCEC, exchangeable Mg^2+^ and pH were the most important predictors for SOC content, while the SOC content, exchangeable Mg^2+^ and exchangeable Ca^2+^ had the most explanatory power for SIC content (Figure 4). The results predicted from the Random Forest model were highly corresponding to the measured contents, achieving *R* ^2^_ajusted_ (Adjusted Coefficient of Determination) reached over 0.95 (Figure 4b) and 0.91 (Figure 4d) for SOC and SIC contents, respectively.

### 3.5 The C Balance and Fluxes in the Atmosphere‐Soil‐Effluent System

The C recoveries of all treatments, except for the weathered basalt with straw treatments (86%), were above 90% (Table 1).

**TABLE 1:** Carbon fluxes in percentage within the atmosphere‐soil‐effluent systems.

	CO_2_‐C (%)	SOC (%)	SIC (%)	Effluent DOC (%)	Effluent DIC (%)	Recovery (%)	IC pool gain (SIC + DIC, %)
Control	7.5 ± 0.34d	93 ± 2.5a	1.0 ± 0.74b	0.35 ± 0.01c	0.16 ± 0.01c	102 ± 2.1	1.2 ± 0.37b
+F. Basalt	5.6 ± 0.23f	83 ± 2.5bc	3.3 ± 1.6a	0.44 ± 0.01a	0.40 ± 0.03a	93 ± 1.5	3.7 ± 0.81a
+W. Basalt	6.4 ± 0.07e	85 ± 1.3b	4.4 ± 0.65a	0.32 ± 0.01d	0.13 ± 0.00 cd	97 ± 1.5	4.5 ± 0.33a
+G.W. Basalt	6.0 ± 0.07ef	90 ± 2.2a	4.1 ± 1.3a	0.36 ± 0.01c	0.14 ± 0.01c	101 ± 1.1	4.2 ± 0.65a
+Straw	10 ± 0.24b	83 ± 1.4bc	0.96 ± 0.29b	0.36 ± 0.01c	0.12 ± 0.02de	95 ± 1.7	1.1 ± 0.14b
+F. Basalt & Straw	9.6 ± 0.36c	78 ± 0.46de	0.90 ± 0.42b	0.40 ± 0.02b	0.22 ± 0.01b	90 ± 0.26	1.1 ± 0.21b
+W. Basalt & Straw	11 ± 0.27a	74 ± 1.5e	0.91 ± 0.44b	0.33 ± 0.01 cd	0.09 ± 0.00e	86 ± 1.5	1.0 ± 0.22b
+G.W. Basalt & Straw	11 ± 0.23a	81 ± 0.80 cd	0.87 ± 0.60b	0.34 ± 0.01c	0.08 ± 0.01e	92 ± 0.59	0.95 ± 0.30b

*Note:* It is depicted as mean ± SE for all treatments after 6 months of incubation period. Data were corrected for dilution effect. The ‘IC gain’ is the of SIC and DIC. The fresh basalt, weathered basalt and ground‐weathered basalt are referred to as ‘F. Basalt’, ‘W. Basalt’ and ‘G.W. Basalt’ in the table, respectively. Lowercase letters indicate significant differences between treatments (*p* < 0.05).

In the effluent, fresh basalt treatments showed significant increases in DOC, DIC, effluent pH and dissolved Mg. In contrast, ground‐weathered basalt treatments showed slight increases in DOC but reductions in dissolved Mg and Ca (Figure S5). Fresh basalt treatments shifted the effluent composition from DOC‐dominated to DIC‐dominated, with DIC content trending upward over time during the 6 months of incubation period. However, weathered basalt and ground‐weathered basalt treatments did not show any DIC increase compared to the control soils (Figure S5).

The contribution of SIC to the total C in the system was significantly higher in fresh basalt (~4%), weathered basalt (~4%) and ground‐weathered basalt (~4%) treatments compared to control soils (~1%), as shown in Table 1. However, SOC contribution was significantly reduced in fresh basalt and weathered basalt treatments but remained unchanged in ground‐weathered basalt treatments compared to the control soil (Table 1). Combined with the contribution of DIC in the effluent, the formation of IC pool (SIC+DIC) was around 4% in fresh basalt, weathered basalt, and ground‐weathered basalt treatments, significantly higher than the control soil (Table 1). These resulted in a net total C loss (SOC losses + IC gain) from the system to be approximately 6%, 13%, 10%, and 6% in the control soil, fresh basalt treatments, weathered basalt treatments, and ground‐weathered treatments, respectively (Table 1).

With the straw addition, CO_2_ emissions increased significantly, reaching ~10%–11% of the C in the system (Table 1). The contribution of DOC and DIC in the effluent remained below 1%, although significantly higher DIC content and contribution were observed in fresh basalt treatments combined with straw (Figure S5, Table 1). Both the fresh basalt treatments and fresh basalt combined with straw treatments significantly increased DIC to a similar contribution to the C in the system, but the absolute amount of DIC was significantly higher in fresh basalt treatments compared to those combined with straw (Figure S5, Table 1).

SIC contribution in fresh basalt, weathered basalt and ground‐weathered basalt treatments decreased from ~3% to 4% to < 1% after straw addition (Table 1). The ground‐weathered basalt treatments with straw maintained SOC contributions similar to only straw addition treatments (Table 1).

## 4 Discussion

### 4.1 Enhanced SIC and DIC Accrual May Offset Initial SOC Losses due to Rising pH

Both fresh basalt and its combination with straw significantly increase soil pH (Figure 2a), primarily due to rapid free H^+^ neutralization during olivine dissolution. Concurrently, fresh basalt causes a significant reduction in SOC content (Figure 1b, Table 1), likely due to the rising pH, which promotes solubilization of SOM (Marinos and Bernhardt 2018), stimulates microbial respiration (Wang and Kuzyakov 2024; Zhang et al. 2020) and accelerates SOM decomposition rate (Wang and Kuzyakov 2024; Yan et al. 2023; Zhang et al. 2020). Our findings align with previous studies reporting accelerated SOC mineralization following the application of ERW materials other than basalt (Haque et al. 2019; Khalidy et al. 2023; Yan et al. 2023). However, the rising pH‐induced effects of ERW materials vary across studies, potentially influenced by the initial acidity of soils and types of ERW materials used (Buss, Hasemer, Sokol et al. 2024; Khalidy et al. 2023). Generally, acidic soils are more prone to SOC losses driven by alkalinity compared to neutral and alkaline soils.

Rising pH observed in fresh basalt treatments diminishes at a more advanced weathering stage, as observed in weathered basalt and ground‐weathered basalt treatments (Figure 2a). This marginal pH change occurs due to slower dissolution rates of less‐reactive minerals such as feldspar and pyroxene following the depletion of olivine. Consequently, the pH‐induced SOC losses after 6 months of incubation in these treatments are significantly reduced, resulting in comparable SOC content to the control soils in ground‐weathered basalt treatments (Figure 1b, Table 1). Differences in SOC contents among treatments are correlated with soil pH, eCEC and the relative abundance of exchangeable Ca^2+^ and Mg^2+^ at two weathering stages (Figure 4a), which further buffer the pH in the soil solution.

Promoted H^+^ neutralization during olivine dissolution also increases bicarbonate alkalinity (Oelkers et al. 2018) and soluble Mg^2+^ in soil solution, which enhances the dissolution of CO_2_ into soil solution forming bicarbonate (the primary form of DIC in this pH range) (Wu et al. 2024). Combined with the reaction of carbonic acid with olivine forming bicarbonate, these processes lead directly to a significantly higher DIC concentration in the effluent observed in fresh basalt treatments (Table 1, Figure S5), suggesting high potential for IC sequestration through transporting to groundwater and ocean systems. An increase in effluent pH in fresh basalt treatments further supports this pathway (Figure S5). In contrast, weathered basalt and ground‐weathered basalt treatments exhibit no significant changes in the effluent DIC concentration or in the pH relative to the control soils (Table 1, Figure S5) as marginal pH changes in these treatments have minor impacts on the shift of carbonic acid equilibrium for bicarbonate formation. However, the released soluble Ca^2+^ may enhance bicarbonate alkalinity by facilitating CO_2_ hydration and H^+^ transfer processes (Wu et al. 2024).

A significant SIC accumulation is observed in fresh basalt, weathered basalt, and ground‐weathered basalt treatments (Figure 1c). This accrual can be attributed to the increased soil pH, elevated DIC concentrations in the soil solution, and their enhanced association with soluble Mg^2+^ and Ca^2+^ derived from mineral dissolution, as indicated by increasing exchangeable Mg^2+^ and Ca^2+^ in soils (Figure 4c) and elevated soluble Mg^2+^ and Ca^2+^ in the effluent (Figure S5), which is necessary for the formation of pedogenic carbonate. Fresh basalt releases large amounts of soluble Mg^2+^ due to the preferential dissolution of Mg‐rich olivine, resulting in a higher exchangeable Mg^2+^. In contrast, ground‐weathered basalt releases more Ca^2+^ due to the depletion in olivine, as evidenced by higher exchangeable Ca^2+^ (Figure 2c), low olivine peaks in XRD analyses (Figure S1) and a significant reduction in total Mg and Fe contents (Table S1). Consequently, the slower dissolution of less‐reactive Ca‐rich minerals (such as feldspars) via carbonic acid dissociation reaction and marginal pH change limit the rapid DIC accumulation in the soil solution, thereby prioritizing SIC formation via Ca‐carbonate precipitation in weathered basalt and ground‐weathered basalt treatments. Thus, weathered basalt and ground‐weathered basalt treatments show significant SIC accumulation. These slower‐dissolving Ca‐rich minerals via carbonic acid can gradually become more critical for SIC accrual at advanced weathering stages, explaining their sustained SIC contribution despite their depletion in olivine.

Therefore, our findings demonstrate that the IC pool derived from SIC accrual occurs at both olivine‐enriched and ‐depleted stages, while DIC accumulation in the effluent only happens at the initial weathering stage (fresh basalt treatments). The long‐term IC accrual by sustained SIC accrual throughout both weathering stages may provide the potential to offset or even exceed the initial SOC losses induced by rising soil pH in the long term.

### 4.2 Higher New OM Retention by Weathered Basalt Compensates for the SIC Accrual Reduction Induced by Straw Decomposition

Straw addition acidifies the soil and limits the formation of SIC in all treatments (Figures 1b,c and 4c). The additional OC and N from straw stimulate the decomposition of SOM, thereby increasing free H^+^ activity via organic acid production (Macias‐Benitez et al. 2020) and nitrification processes (Bolan et al. 1991). These additional free H^+^ partially offset the neutralization of free H^+^ during olivine dissolution, thus reducing the pH increase in fresh basalt treatments with straw compared to those without straw. Differently from the H^+^ released via the dissociation of carbonic acid, which is the key process for carbon dioxide removal, this stronger acid‐derived H^+^ can compete with carbonic acid for olivine dissolution (Guo et al. 2015). Combined with the reduced bicarbonate alkalinity, they thereby limit the accumulation of DIC, which directly causes a lower DIC concentration in the effluent of fresh basalt treatments with straw (Table 1, Figure S5). This also directly diminishes the SIC formation due to the limited DIC available for the association with Ca^2+^ and Mg^2+^. Consequently, no significant differences in SIC contents at both weathering stages were observed relative to the control soils (Figure 1c).

Under the same dosage of straw application, fresh basalt and ground‐weathered basalt treatments retain similar amounts of native SOC as the control soils (Figure 3b), resulting in comparable SOC contents. In contrast, weathered basalt treatments show the weakest retention capability for native SOC (Figure 3b). This discrepancy is likely due to the coarser texture of weathered basalt that hinders the weathering rates and mineral surface effectiveness (less exchangeable cations; Table S1), as it contains over 60% of sand‐sized particles. The physical grinding process reduces these sand‐sized particles to silt size in ground‐weathered basalt (Table S1). A similar texture of fresh basalt and ground‐weathered basalt results in a comparable capability to retain native SOC as the control soils.

Interestingly, although fresh basalt has a significantly higher SSA compared to the control soils (Table S1), it does not enhance the stabilization of straw‐derived OC (Figure 3a). Indeed, no significant SSA increase is detected once fresh basalt is mixed with the control soils (Tables S2, Table S3). In contrast, ground‐weathered basalt, despite having a similar particle size to fresh basalt, has a significant enhancement in the stabilization of straw‐derived OC compared to the control soils (Figure 3a), likely because of its increased SSA (roughly four times higher SSA compared to the control soils; Table S2, Table S3) and the shifts in cation dominance from Mg^2+^ to Ca^2+^ following the depletion of olivine, indicated by lower soluble Mg^2+^ in the effluent and higher exchangeable Ca^2+^. The Ca^2+^, with its higher flocculating power and smaller hydration radius (Rao and Mathew 1995), may form stronger cation bridges that stabilize SOC more effectively (Amézketa 1999; Rengasamy et al. 1986), whereas the Mg^2+^‐dominance may have a weaker or even an adverse impact on soil structure due to its larger hydration radius that may inhibit the aggregation formation (Emerson and Chi 1977; Zhang and Norton 2002).

Thus, straw addition acidifies the soil by generating additional H^+^ during its decomposition, which partially offsets the neutralization of free H^+^, reduces bicarbonate alkalinity, and competes with carbonic acid for olivine dissolution. Consequently, it limits the DIC formation in soil solution, thereby leading to lower DIC concentrations in the effluent and limiting its accessibility for the associations with Ca^2+^ and Mg^2+^ forming SIC. However, enhanced stabilization of straw‐derived new OC by ground‐weathered basalt due to its higher SSA and exchangeable Ca^2+^ can partially compensate for the reduced IC pool resulting from straw decomposition.

### 4.3 Soil C Fluxes Are Distinctly Determined by Basalt Weathering Stages and Plant Residues Inputs

Applying basalt at various weathering stages influences not only TC content but also C fluxes through effluent and atmospheric CO_2_ release. After 6 months of incubation period, over 75% of C is retained in the soil. The majority of C retained in soil (> 95%) is in organic form. Only a minor fraction (< 1%) of C is leached (Table 1), of which DIC accounts for a maximum of ~45% in fresh basalt treatments. Hence, ERW‐induced contributions to C sequestration primarily affect SOC content, given that IC gain (SIC + DIC) constitutes < 5% of C fluxes (Table 1).

**FIGURE 5:**
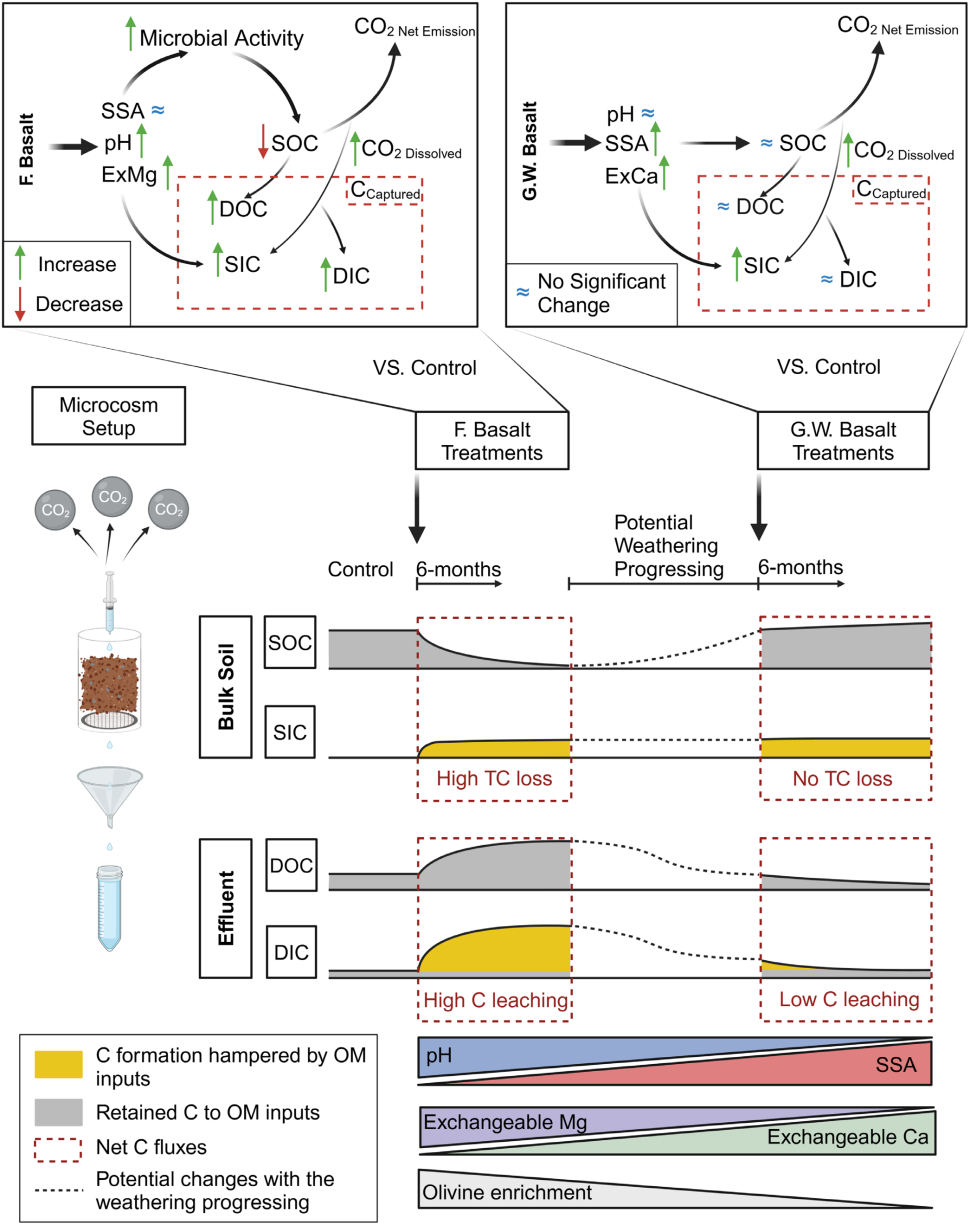
Schematic illustration of carbon fluxes and cycling within soil, atmosphere and effluent. Fresh basalt and ground‐weathered basalt treatments are compared to the control soils. The time‐diagram shows changes in soil organic carbon (SOC), soil inorganic carbon (SIC), dissolved inorganic carbon (DIC) and dissolved organic carbon (DOC), as influenced by basalt weathering processes, relative to control soils. Associated changes in soil physicochemical properties and olivine enrichment across weathering stages are also indicated. The ‘ExCa’, ‘ExMg’ and ‘SSA’ are referred to ‘exchangeable Ca^2+^’, ‘exchangeable Mg^2+^’ and ‘specific surface area’, respectively.

While other studies have reported substantial DIC sequestration through DIC leaching with the application of fresh ERW materials (Manning et al. 2024), our findings suggest this condition may only occur at the initial weathering stage (~0.4%; Table 1 and Figure 5) as ground‐weathered basalt treatments (olivine‐depleted fine‐sized weathered basalt) significantly reduce DIC leaching to levels comparable to control soils (~0.14%; Table 1 and Figure S5). The observed marginal change in pH, reduced exchangeable Mg^2+^ and slower carbonic acid reaction due to olivine depletion likely limit the DIC formation at more advanced weathering stages (Figure S5). Instead, increased soluble Ca^2+^ from ground‐weathered basalt treatments (Figure 2 and Figure S5) prioritizes the formation of SIC rather than contributing to additional DIC in the effluent.

Carbon losses can be partially captured as SIC and DIC leaching (Table 1) after 6 months of incubation. Fresh basalt treatments promote DIC leaching (~0.4%) and enhance the SIC accrual (~4%) compared to the control soils, though it still results in ~7% more, equal to approximately 3 t ha^−1^ (0–30 cm) more net C loss from the soil in the first half year compared to the control soils. In contrast, ground‐weathered basalt treatments retain both SOC and SIC, resulting in 0 ~ 0.5 t ha^−1^ (0–30 cm) less net C losses within soils in the first half year than the control soils. This indicates that the capability of C retention in the soil increases with the weathering progressing, primarily due to reduced pH‐induced SOC losses, which can be moderated by soil acidification via straw decomposition (Figure 2). Consequently, when the straw is added once during the first half year, although CO_2_ emissions rise (Figure S4) and both SIC accumulation and DIC formation decrease, net C losses remain lower due to reduced pH‐induced native SOC losses, resulting in comparable net C losses of fresh basalt and ground‐weathered basalt treatments to the control soils (Table 1).

Therefore, the impact of basalt applications on soil C turnover and fluxes varies with the stages of basalt weathering (Figure 5). During the initial weathering stage (fresh basalt treatments), olivine dissolution induces the increased soil pH and eCEC, provides, and promotes additional soluble Mg^2+^. These effects tend to promote microbial activity to OM decomposition, thus releasing more DOC to the effluent (Figure 5). Meanwhile, combined with free H^+^ neutralization and carbonic acid dissociation during olivine dissolution, the increasing bicarbonate alkalinity and formation of bicarbonate result in a higher concentration of DIC in soil solution, which directly increases DIC leaching and enhances the association with Mg^2+^ forming SIC (Figure 5). As weathering progresses to olivine‐depletion stages (ground‐weathered basalt treatments), the mechanism of C losses changes due to the slower free H^+^ neutralization and cation releasing. The slower reaction of carbonic acid with less‐reactive minerals limits the DIC accumulation as much as those in fresh basalt treatments, thereby resulting in the limited formation of SIC association with Ca^2+^ without the excess DIC leaching to the effluent (Figure 5). At this stage, higher SSA and more exchangeable Ca^2+^ become critical in reducing SOC losses by enhancing the stabilization of SOC.

Plant residue inputs (straw addition) can distinctly alter C fluxes, mainly by reducing SIC and DIC formation via producing additional free H^+^ through OM decomposition, thereby acidifying soil solutions and reducing bicarbonate alkalinity (Figure 5). This extra H^+^ release also inhibits DIC formation via the competing reaction with carbonic acid for minerals. Consequently, it limits SIC formation by decreasing the availability of DIC for reaction with Ca^2+^ and Mg^2+^ at both weathering stages. As a result, straw addition significantly reduces IC gains at both weathering stages.

Consequently, the interplay between basalt weathering stages and plant‐derived OM inputs creates distinctly different patterns of C. While initial weathering stages (fresh basalt) demonstrate strong DIC and SIC formation accompanied by significant SOC losses, advanced weathering stages (ground‐weathered basalt) maintain stable soil pH and SIC formation and promote both native and straw‐derived OC stabilization due to increased SSA and Ca^2+^ dominance, leading to less net C losses after 6‐month incubation.

These findings provide an essential SOC‐based solution for carbon sequestration associated with ERW and underscore the importance of considering both IC and OC when evaluating the effectiveness of ERW for long‐term carbon sequestration. The OC pathway is particularly relevant in slightly acidic and acidic soils in temperate and tropical zones with abundant precipitation and suitable temperatures that continuously receive biomass inputs, thus partially constraining the DIC formation. With the depletion of Mg‐enriched silicates (e.g., olivine for basalt) at more advanced weathering stages, these advanced weathered ERW materials can stabilize soil pH and provide diverse exchangeable cations (depending on weathering stages and ERW materials) and higher SSA to enhance soil capacity for retaining newly added OM and minimizing native SOC losses, thereby securing the long‐term benefits of ERW for carbon sequestration via both IC and OC pathways.

## 5 Conclusion

This study demonstrates that different weathering stages of basalt significantly influence soil C turnover and fluxes in temperate cropland topsoil. Over 6 months of incubation, both fresh basalt (fresh basalt treatments) and weathered basalt (weathered basalt and ground‐weathered basalt treatments) increase SIC accumulation. Fresh basalt promotes the release of Mg^2+^ due to olivine enrichment, while weathered basalt shifts to Ca^2+^ dominance as olivine depletes with weathering progressing.

Fresh basalt increases soil pH via rapid free H^+^ neutralization, releases additional soluble Mg^2+^ during olivine dissolution, and increases bicarbonate alkalinity in soil solution. Combined with the reaction of carbonic acid with olivine, they synergistically promote DIC formation (~0.4%), thereby enhancing the SIC accumulation (~4%). However, it also triggers significantly higher SOC losses (~17%) compared to the control soil (~7%) after 6‐month incubation. In contrast, weathered basalt (olivine‐depleted) does not change the pH of the soil solution due to slower free H^+^ neutralization of less‐reactive mineral dissolution following olivine depletion, resulting in SOC losses comparable to the control soils (~7%). The additional soluble Ca^2+^ and dissociation of carbonic acid reacting to these minerals also promote the SIC accrual (~4%). Additionally, this weathered basalt (ground‐weathered basalt treatments) demonstrates higher SOC retention capabilities for both native OC and straw‐derived new OC, likely attributed to increased specific surface area and Ca‐mediated OC stabilization.

Although basalt‐induced SIC accumulation provides clear benefits for soil carbon sequestration in bare soils at both fresh and weathered stages, our findings indicate that the presence of continuous plant residue inputs modifies this outcome. Specifically, plant residues release additional free H^+^ during decomposition, acidifying soils and thereby partially suppressing DIC formation due to decreased bicarbonate alkalinity and the competitive reaction of free H^+^ with carbonic acid for mineral dissolution. Consequently, both SIC accrual and effluent DIC concentrations decrease compared to those without plant inputs. Therefore, in soils with continuous plant residue inputs, like most soils under agricultural production, the effectiveness assessment of ERW should also take these new OM inputs as one of the essential factors. The long‐term climate mitigation benefits of ERW can significantly depend on SOC loss reduction and new OC retention facilitated by weathered ERW materials (e.g., ground‐weathered basalt) besides the IC formation.

Thus, our findings demonstrate that maximizing the effectiveness of ERW also requires prioritizing SOC stabilization, particularly in systems with continuous OM inputs, as evidenced by the superior retention in ground‐weathered basalt treatments. This indicates that the ERW materials may be a substitution for lime in agricultural systems requiring soil acidity correction (e.g., tropical agriculture), due to their significant ability for soil acidity correction at the initial weathering stage and enhanced carbon sequestration at the advanced weathering stage. Special attention should be paid to the interactions of OC and IC pools associated with ERW in the long term, which can determine its applicability for mitigating climate change. Further research is needed to optimize ERW application strategies and models tailored to varying soils and other environmental conditions, considering distinct C fluxes at varying weathering stages and new OM inputs.

## Author Contributions

**Kaiyu Lei:** conceptualization, data curation, formal analysis, investigation, methodology, validation, visualization, writing – original draft, writing – review and editing. **Franziska B. Bucka:** conceptualization, investigation, methodology, project administration, supervision, writing – review and editing. **Pedro P. C. Teixeira:** conceptualization, investigation, methodology, supervision, writing – review and editing. **Franz Buegger:** data curation, writing – review and editing. **Christopher Just:** investigation, writing – review and editing. **Ingrid Kögel‐Knabner:** conceptualization, funding acquisition, investigation, project administration, supervision, validation, visualization, writing – review and editing.

## Conflicts of Interest

The authors declare no conflicts of interest.

## Supporting information


Data S1.

## Data Availability

The data that support the findings of this study are openly available in Zenodo at https://doi.org/10.5281/zenodo.15127645.
