# Supplementary material for: Balancing Organic and Inorganic Carbon Dynamics in Enhanced Rock Weathering: Implications for Carbon Sequestration
Source: Glob Chang Biol. 2025 Apr 16;31(4):e70186. doi: 10.1111/gcb.70186 (PMC12001311; doi:10.1111/gcb.70186)
Supplement: Supplementary file 1 — Data S1. [file GCB-31-e70186-s001.docx]

**Balancing Organic and Inorganic Carbon Dynamics in Enhanced Rock Weathering: Implications for Carbon Sequestration**

Kaiyu Lei ^a^, Franziska B. Bucka ^a b^, Pedro P.C. Teixeira ^a^, Franz Buegger ^c^, Christopher Just ^a^, Ingrid Kögel-Knabner ^a d^

^a^ Chair of Soil Science, School of Life Science, Technical University of Munich, Emil-Ramann-Straße 2, 85354 Freising, Germany

^b^ Soil Geography and Ecosystem Research, Institute of Physical Geography, Goethe University Frankfurt, Altenhöferallee 1, 60438 Frankfurt am Main, Germany

^c^ Research Unit Environmental Simulation, Helmholtz Zentrum München, German Research Center for Environmental Health, 85764 Neuherberg, Germany

^d^ Institute for Advanced Study, Technical University of Munich, Lichtenbergstraße 2a, 85748 Garching, Germany

**Supplementary materials**


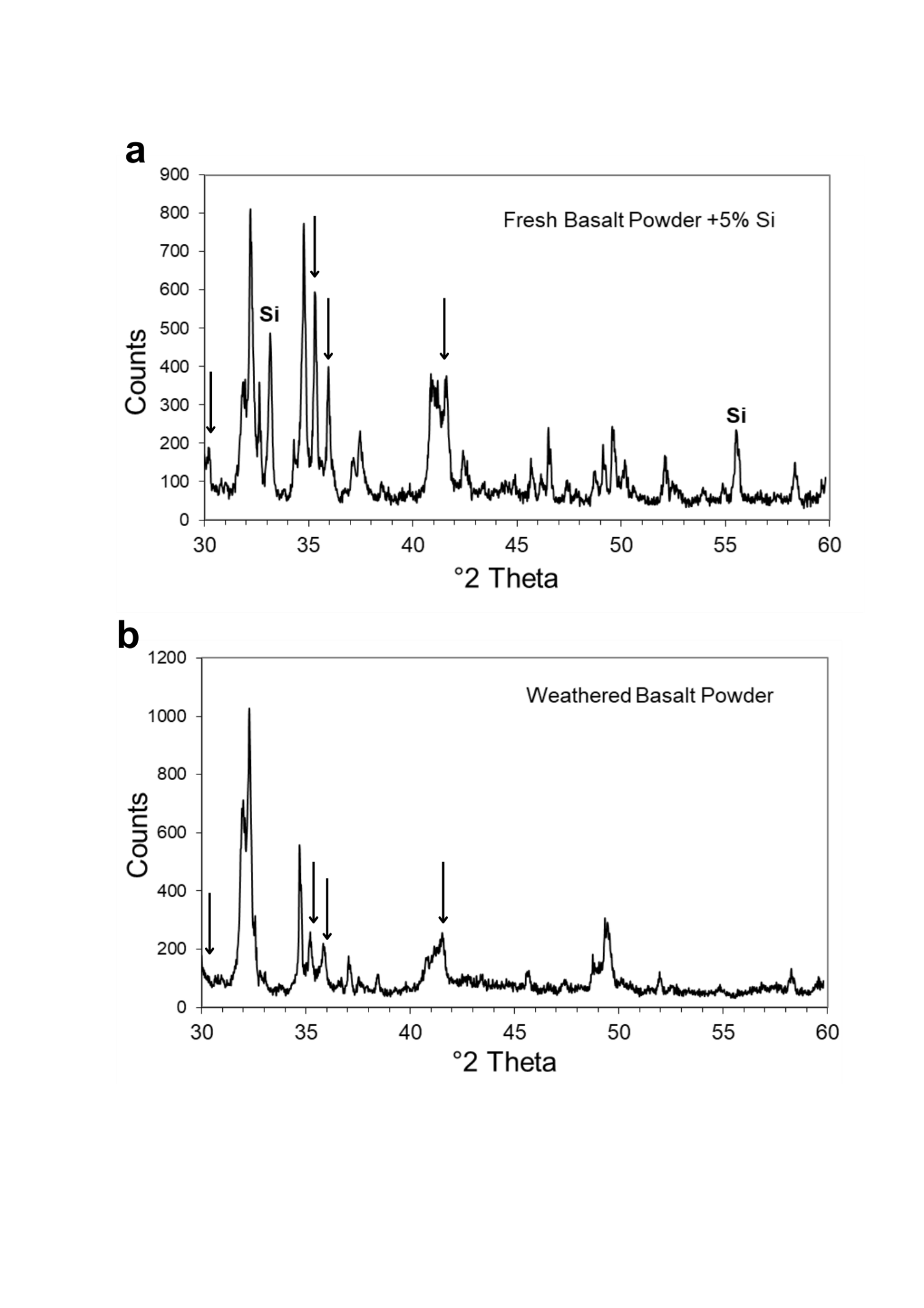


**Fig. S1** XRD results of a) fresh basalt powder + 5% silicates, and b) the weathered basalt powder. Both materials are derived from the same quarry. The typical peaks representing olivine minerals are marked with arrows.

**Table S1** The physicochemical properties of materials used for incubation experiment. Data are mean, n = 3 as technical replicates.

|  | Cropland Soil | Fresh Basalt | Weathered Basalt (W. Basalt) | Ground Weathered Basalt (G.W. Basalt) | Rice Straw |
| --- | --- | --- | --- | --- | --- |
| pH (0.01 M CaCl2) | 5.8 | 7 | 6.7 | 6.7 | / |
| Sand/Silt/Clay | 31/51/18 | 29/70/1 | 63/26/11 | 24/64/12 | / |
| BET (m^2^ g^-1^) | 8.1 | 15 | 34 | 35 | / |
| TC (mg g^-1^) | 17 | 4.4 | 0.68 | 0.69 | 404 |
| OC (mg g^-1^) | 16.8 | 0.16 | 0.66 | 0.64 | 404 |
| TN (mg g^-1^) | 1.6 | 0.01 | 0.06 | 0.07 | 29 |
| Total Ca (mg g^-1^) | 4.9 | 64 | 48 | 49 | / |
| Total Mg (mg g^-1^) | 4.9 | 60 | 17 | 18 | / |
| Total Al (mg g^-1^) | 65 | 64 | 83 | 86 | / |
| Total Fe (mg g^-1^) | 23 | 74 | 62 | 63 | / |
| Total P (mg g^-1^) | 0.94 | 4.1 | 6.2 | 6.3 | / |
| Total Cr (mg g^-1^) | 0.04 | 0.26 | 0.05 | 0.05 | / |
| Total Ni (mg g^-1^) | < 0.01 | 0.29 | 0.03 | 0.03 | / |
| δ^13^C (‰ V-PDB) | -27.71 | -2.66 | -24.42 | -25.49 | 238 |
| δ^15^N (‰ vs.air-N_2_) | 7 | / | / | / | 1375 |

* The G.W. Basalt was grinded to a similar particle size distribution as F. Basalt to exclude the impact of texture, and to better simulate the weathering of F. Basalt and the weathering of W. Basalt.

**Table S2** The initial physiochemical properties of all treatments. Data was corrected for dilution effect. Data are mean, n = 3 as technical replicates. The fresh basalt, weathered basalt and ground weathered basalt treatments are referred to as ‘F. Basalt’, ‘W. Basalt’, and ‘G.W. Basalt’ in the table, respectively.

|  | Control | + F. Basalt | + W. Basalt | + G.W. Basalt | + Straw | + F. Basalt & Straw | + W. Basalt & Straw | + G.W. Basalt & Straw |
| --- | --- | --- | --- | --- | --- | --- | --- | --- |
| pH (1:2/5 CaCl2) | 5.8 | 6 | 5.7 | 5.8 | 5.8 | 5.9 | 5.8 | 5.8 |
| BET (m^2^ g^-1^) | 8.1 | 8.4 | 9.2 | 10.7 | 8.1 | 9.2 | 10.7 | 8.9 |
| TC (mg g^-1^) | 17 | 16.9 | 17 | 17 | 21 | 21 | 20.9 | 21 |
| OC (mg g^-1^) | 16.8 | 16.7 | 16.8 | 16.7 | 21 | 20.9 | 20.8 | 20.8 |
| TN (mg g^-1^) | 1.6 | 1.58 | 1.6 | 1.62 | 1.9 | 1.85 | 1.86 | 1.86 |
| Total Ca (mg g^-1^) | 4.9 | 7.1 | 6.4 | 6.7 | 4.8 | 7.1 | 6.4 | 6.7 |
| Total Mg (mg g^-1^) | 4.9 | 7.5 | 5.5 | 5.8 | 5.2 | 7.6 | 5.5 | 5.7 |
| Total Al (mg g^-1^) | 65 | 65 | 66 | 67 | 65 | 63 | 65 | 66 |
| Total Fe (mg g^-1^) | 23 | 26 | 26 | 26 | 24 | 25 | 25 | 26 |
| Total P (mg g^-1^) | 0.94 | 1.1 | 1.2 | 1.2 | 0.96 | 1 | 1.2 | 1.2 |
| Total Cr (mg g^-1^) | 0.04 | 0.04 | 0.04 | 0.04 | 0.04 | 0.04 | 0.04 | 0.04 |
| Total Ni (mg g^-1^) | < 0.01 | 0.02 | 0.02 | 0.02 | 0.01 | 0.02 | 0.02 | 0.02 |
| δ^13^C (‰ V-PDB) | -27.71 | -27.63 | -27.94 | -27.93 | 2.52 | 17.4 | 17.8 | 17.6 |
| δ^13^C (‰ V-PDB, IC removed) | -27.72 | -27.64 | -27.82 | -27.86 | -2.86 | 15.19 | 20.9 | 19.16 |

**Table S3** The physical properties of all treatments after six months of incubation (SOM not removed). Data are mean ± SE, n = 5. The fresh basalt, weathered basalt and ground weathered basalt treatments are referred to as ‘F. Basalt’, ‘W. Basalt’, and ‘G.W. Basalt’ in the table, respectively.

|  | Control | + F. Basalt | + W. Basalt | + G.W. Basalt | + Straw | + F. Basalt & Straw | + W. Basalt & Straw | + G.W. Basalt & Straw |
| --- | --- | --- | --- | --- | --- | --- | --- | --- |
| BET (m^2^ g^-1^) | 8.6 ± 0.17 | 8.6 ± 0.08 | 10.3 ± 0.35 | 9.49 ± 0.29 | 8.53 ± 0.26 | 8.22 ± 0.29 | 9.47 ± 0.15 | 9.48 ± 0.07 |
| Sand (%) | 29 ± 1 | 31 ± 1 | 33 ± 1 | 30 ± 2 | 28 ± 2 | 34 ± 3 | 31 ± 1 | 27 ± 1 |
| Silt (%) | 41 ± 1 | 41 ± 1 | 40 ± 1 | 43 ± 1 | 41 ± 1 | 38 ± 2 | 41 ± 1 | 42 ± 1 |
| Clay (%) | 29 ± 0.5 | 28 ± 0.4 | 28 ± 0.2 | 28 ± 0.6 | 29 ± 0.2 | 26 ± 0.5 | 28 ± 0.58 | 28 ± 0.4 |
| δ^13^C (‰ V-PDB) | -27.88 ± 0.02 | -27.61 ± 0.02 | -27.78 ± 0.03 | -27.91 ± 0.01 | -15.1 ± 0.64 | -15.66 ± 0.44 | -14.42 ± 0.54 | -13.43 ± 0.29 |
| δ^13^C (‰ V-PDB, IC removed) | -27.75 ± 0.06 | -27.59 ± 0.02 | -27.66 ± 0.02 | -27.78 ± 0.08 | -15.27 ± 0.54 | -15.94 ± 0.56 | -14.58 ± 0.57 | -13.72 ± 0.30 |


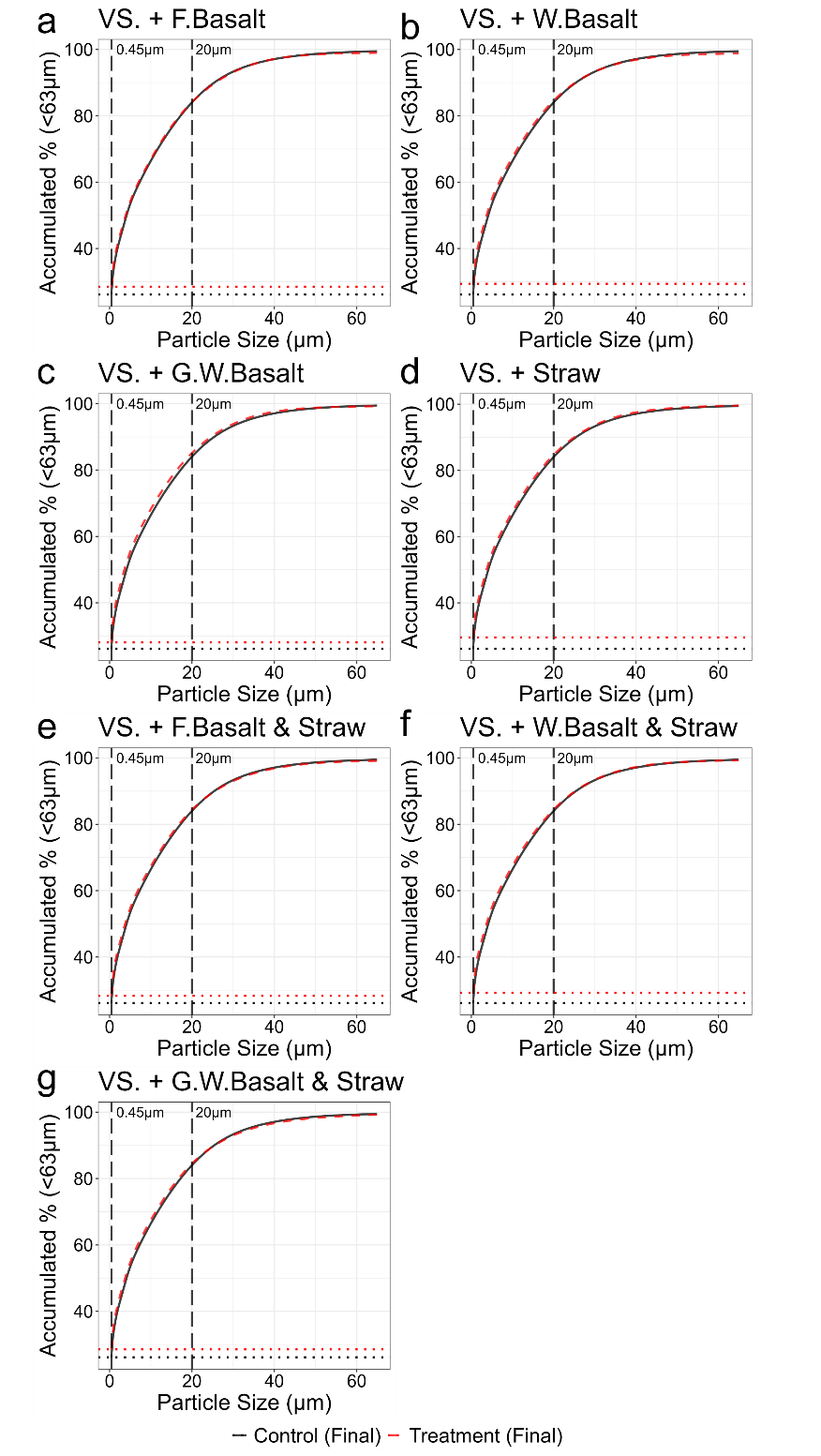


**Fig. S2** The particle size comparison of the control soils with other treatments after six months of incubation. Data are the mean of replicates, n = 5. The fresh basalt, weathered basalt and ground weathered basalt treatments are referred to as ‘F. Basalt’, ‘W. Basalt’, and ‘G.W. Basalt’ in the figure, respectively.


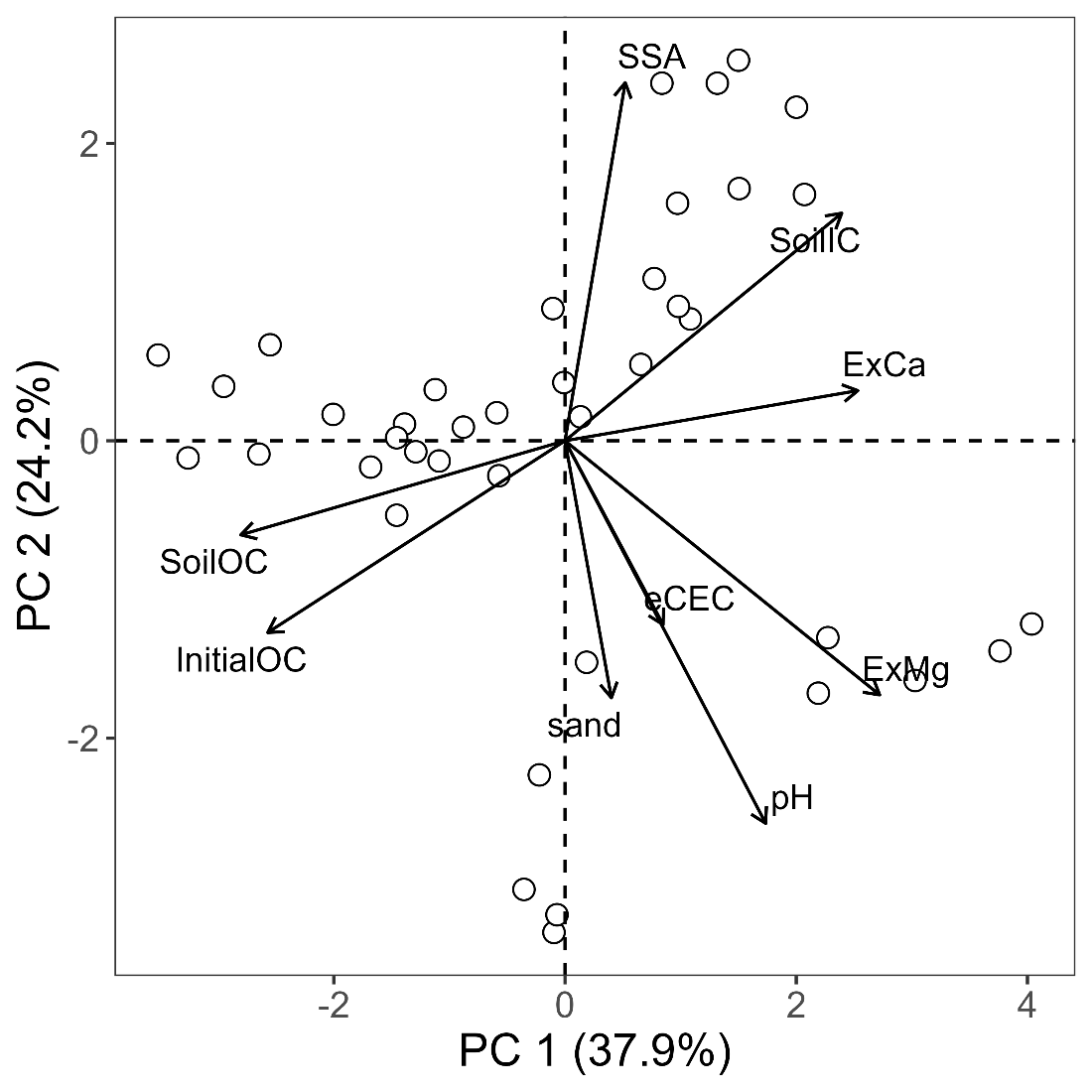


**Fig. S3** The PCA analysis of soil C and other related variables.

**Table S4** The Random Forest model parameters for SOC and SIC in Fig. 4.

|  | Random Forest for SOC | Random Forest for SIC |
| --- | --- | --- |
| OOB error | 0.24 | 0.04 |
| RMSE | 0.23 | 0.01 |
| R^2^ | 0.94 | 0.89 |
| Adj. R^2^ | 0.95 | 0.91 |


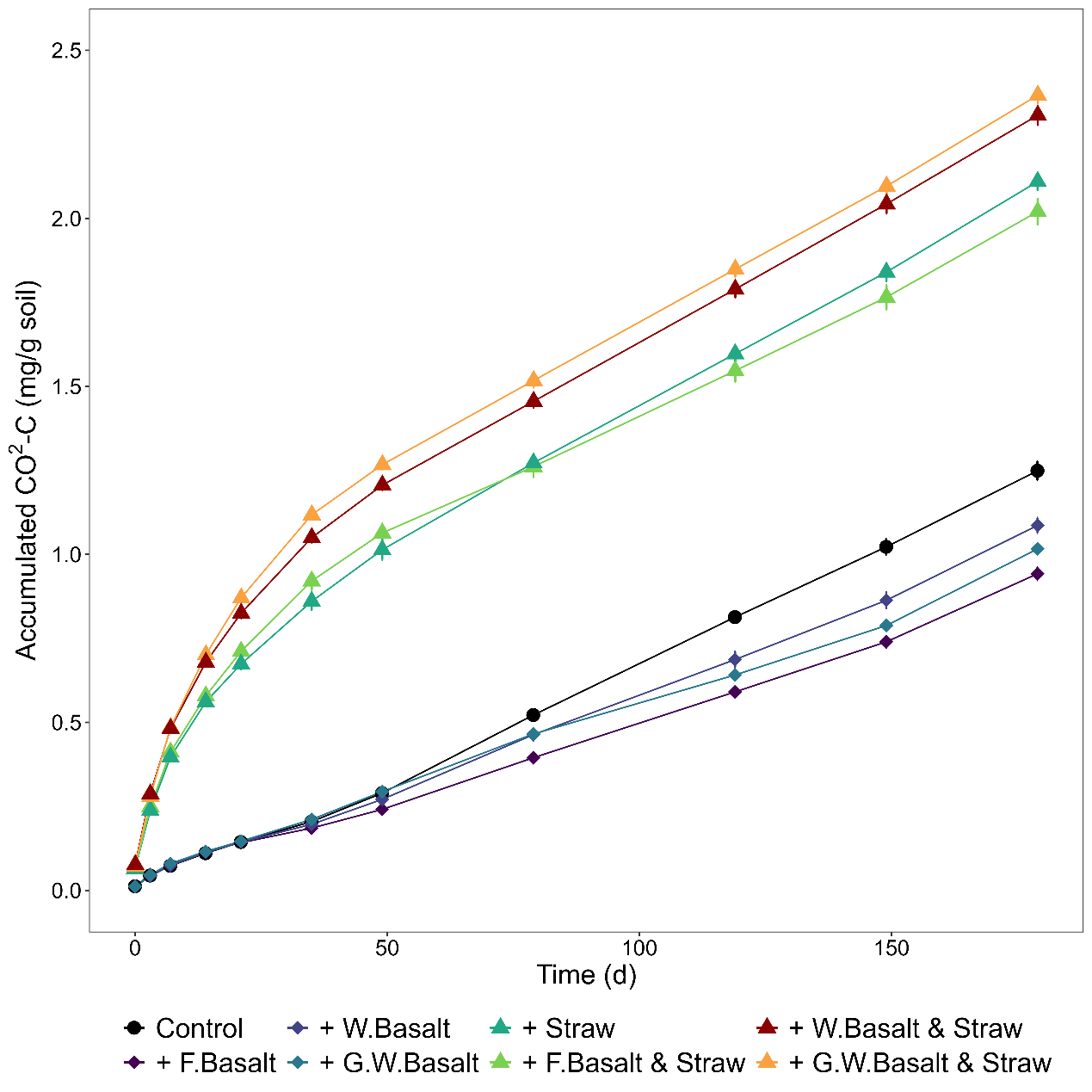


**Fig. S4** The accumulated emission of CO_2_-C from soils during six months of incubation. Data was corrected for dilution effect. Data are mean ± SE, n = 5. The fresh basalt, weathered basalt and ground weathered basalt treatments are referred to as ‘F. Basalt’, ‘W. Basalt’, and ‘G.W. Basalt’ in the figure, respectively.


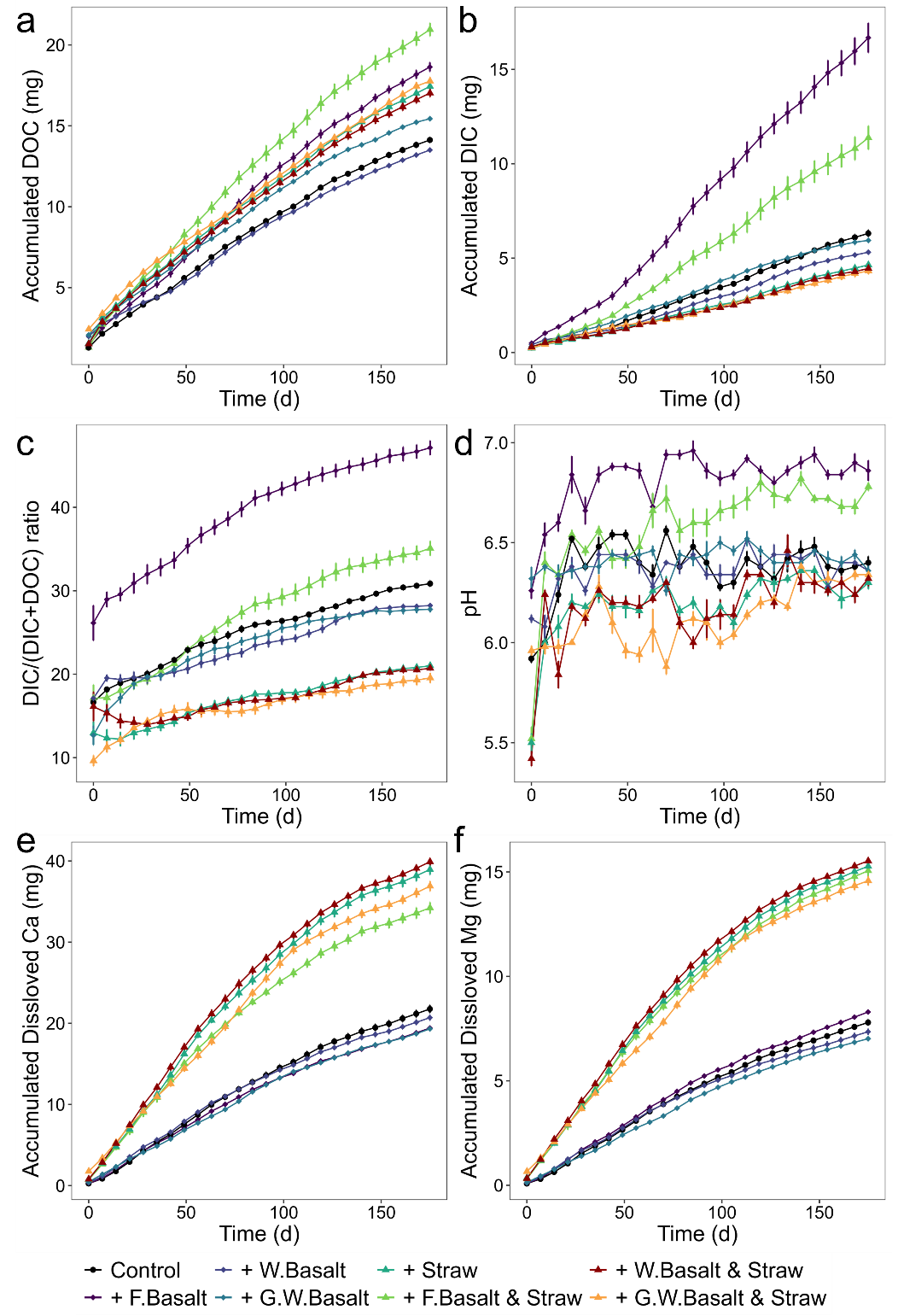


**Fig. S5** The accumulated release of a) DOC; b) DIC; c) DIC to total dissolved C ratio; d) pH; e) dissolved Ca, and f) dissolved Mg in the effluent during six months of incubation. Data was corrected for dilution effect. Data are mean ± SE, n = 5. The fresh basalt, weathered basalt and ground weathered basalt treatments are referred to as ‘F. Basalt’, ‘W. Basalt’, and ‘G.W. Basalt’ in the figure, respectively.


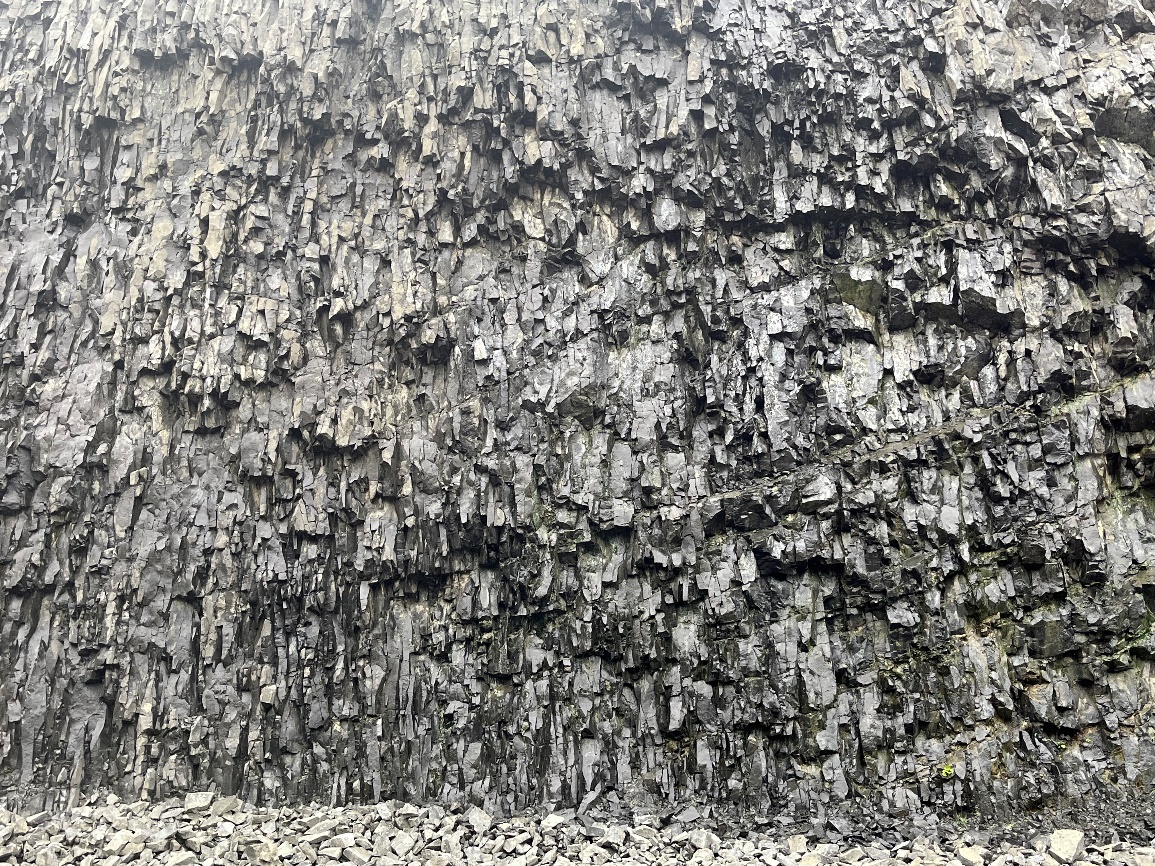

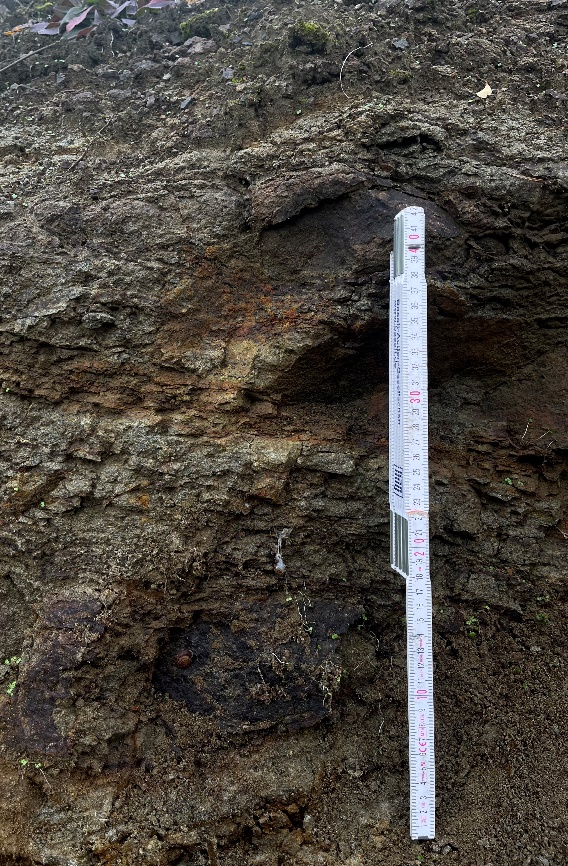


**Fig. S6** The fresh basalt and weathered basalt sampling area in the quarry.
